# Orexin-A Prevents Lipopolysaccharide-Induced Neuroinflammation at the Level of the Intestinal Barrier

**DOI:** 10.3389/fendo.2019.00219

**Published:** 2019-04-10

**Authors:** Lea Tunisi, Nicola Forte, Alba Clara Fernández-Rilo, Isabella Mavaro, Raffaele Capasso, Livia D'Angelo, Nataša Milić, Luigia Cristino, Vincenzo Di Marzo, Letizia Palomba

**Affiliations:** ^1^Department of Veterinary Medicine and Animal Productions, University of Naples Federico II, Naples, Italy; ^2^Endocannabinoid Research Group, Institute of Biomolecular Chemistry (ICB), National Research Council (CNR), Pozzuoli, Italy; ^3^Department of Agricultural Science, University of Naples Federico II, Portici, Italy; ^4^Stazione Zoologica Anton Dohrn, Naples, Italy; ^5^Department of Pharmacy, University of Novi Sad, Novi Sad, Serbia; ^6^Canada Excellence Research Chair on the Microbiome-Endocannabinoidome Axus in Metabolic Health, Faculty of Medicine and Faculty of Agricultural and Food Sciences, Université Laval, Québec City, QC, Canada; ^7^Department of Biomolecular Sciences, University of Urbino Carlo Bo, Urbino, Italy

**Keywords:** gut-brain axis, gut microbiota, orexins, lipopolysaccharides, microglia

## Abstract

In states of intestinal dysbiosis, a perturbation of the normal microbiome composition, the intestinal epithelial barrier (IEB) permeability is increased as a result of the disruption of the epithelial tight junction protein network, in which occludin is mostly affected. The loss of IEB integrity promotes endotoxemia, that is, bacterial lipopolysaccharide (LPS) translocation from the intestinal lumen to the circulatory system. This condition induces an enhancement of pro-inflammatory cytokines, which leads to neuroinflammation through the gut-brain axis. Orexin-A (OX-A), a neuropeptide implicated in many physiological functions and produced mainly in the brain lateral hypothalamic area, is expressed also in several peripheral tissues. Orexin-producing neurons have been found in the myenteric plexus to project to orexin receptor 1 (OX-1R)-expressing enterocytes of the intestinal villi. In the present study we investigated the protective role of OX-A against LPS-induced increase of IEB permeability and microglia activation in both an *in vivo* and *in vitro* model of the gut-brain axis. By exploiting biochemical, immunocytochemical, immunohistochemical, and functional approaches, we demonstrate that OX-A preserves the IEB and occludin expression, thus preventing endotoxemia and subsequent neuroinflammation.

## Introduction

The intestinal barrier, formed by the mucus layer, epithelial cells, and the underlying lamina propria, allows the uptake of nutrients and water, while being restrictive against pathogenic substances and bacteria. Epithelial cells form a continuous monolayer as they are closely attached to each other by tight junction (TJ) proteins that provide cell-adhesion bonds to preserve the integrity of the intestinal barrier. Dysfunction of this barrier triggers an increase of intestinal permeability, thus facilitating translocation of damaging and pro-inflammatory substances and even pathogens to blood circulation. Commensal microorganisms that constitute the gut microbiota represent one of the main interplayers that maintain the balance of the intestinal barrier during physiological conditions, and disruption of their equilibrium can promote disease (1). In fact, when the intestinal microbiota composition is altered, such as during the “dysbiosis” induced by prolonged use of antibiotics, unbalanced diets and obesity, the permeability of the intestinal epithelial barrier (IEB) increases as a consequence of the disruption of the epithelial TJ protein network, allowing the translocation of bacterial lipopolysaccharide (LPS) into the systemic circulation (2). In healthy conditions, several mechanisms are activated to keep LPS at the intestinal level (3). The TJ network involves several proteins including occludin, whose decreased expression on the cell membrane of enterocytes is associated with IEB dysfunction and increased permeability (4–6). Numerous noxious stimuli, including pathogens, oxidative stress, and pro-inflammatory cytokines, are able to affect IEB integrity through the impairment of occludin function (7, 8). Recent studies have shown that the intestinal microbiota can modulate different biological responses inside and outside the gut, including the innate and adaptive immune response during infection and inflammation at the intestinal surface (9). Commensal bacteria are also able to control neuroinflammation through the gut-brain axis, the bidirectional communication between the central and enteric nervous systems (10). Alterations in the composition of the intestinal microbiota affect the central nervous system, among others, by contributing to the impairment of microglia maturation, differentiation, and function mostly due to LPS-induced oxidative stress (11). Orexin-A (OX-A) (also known as hypocretin-1) was initially characterized as a neuropeptide produced by hypothalamic neurons involved in the homeostatic control of central functions such as feeding, sleep-awakeness cycles (12, 13) and neuroprotection (14). The functions of the neuropeptide are performed by binding to the OX-A receptor 1 (OX-1R), a G-protein-coupled receptor signaling via the phospholipase C-beta cascade (15). The neuroprotective role of OX-A has been suggested to be due to the effect on microglia activation; in different models of cerebral ischemia the neuropeptide is able to prevent the increment of LPS-induced tumor necrosis factor alpha (TNF-α) (16). However, many studies have shown that OX-A and OX-1R are present also in peripheral tissues including the gut (14). OX-A-producing neurons have been found in the myenteric plexus to project to OX-1R-expressing enterocytes of the intestinal villi in the IEB (17), possibly suggesting their role in gut motility and intestinal absorption and/or secretion of nutrients (17). In the present study, we investigated the possibility that OX-A may also exert neuroprotection by preventing LPS-induced intestinal barrier dysfunction. Using both *in vitro* and *in vivo* models, we suggest that OX-A counteracts LPS-induced increase of intestinal permeability, thus preventing LPS translocation from the gut to the brain and microglia activation.

## Materials and Methods

### Cell Culture and Treatments

Primary cortical astrocytes/microglia co-cultures, derived from neonatal 3 or 5 days-old C57BL/6 (Charles River), were obtained as described in Palomba et al. (18). Cells were cultured in Eagle's minimal essential medium supplemented with 10% horse serum and 10% fetal bovine serum (Life Technologies), penicillin (50 units/ml) and streptomycin (50 μg/ml) (Life Technologies) at 37°C in T-75 tissue culture flasks gassed with an atmosphere of 95% air-5% CO_2_. At confluency (12–14 DIV) the flasks were shaken to isolate microglial cells, loosely attached to the astrocytes. Microglia were maintained in astrocyte-conditioned medium and used 24 h after plating. Histochemical staining with Griffonia simplicifolia B4-isolectin fluorescein isothiocyanate-conjugated was used to confirm pure preparations of microglia. At the treatment stage microglia was between 1.5 and 2.0 × 10^5^cells/dish. Caco-2 cells, cultured in Eagle's minimal essential medium (Life Technologies) supplemented with 10% horse fetal bovine serum (Life Technologies), penicillin (50 units/ml) and streptomycin (50 μg/ml) (Life Technologies), at 37°C, were seeded at 5 × 10^5^ cells/well onto transwell insert plates (4.67 cm^2^, 0.4 μm pore size, Falcon). The medium was changed on alternate days for 20–21 days until the cells were fully differentiated (TEER value >1,300 Ω cm^2^). The differentiation of Caco-2 cells was evaluated by morphological analysis and alkaline phosphatase activity *in situ* (data not shown).

As *in vitro* model, we used a combination of Caco-2 cells and primary cortical microglia. For co-culture experiment, the transwell inserts containing Caco-2 cells were added into multiple plate wells containing primary cortical microglia plated on polylysine-coated coverslips. Caco-2 cells were treated to the apical chamber: (i) untreated, (ii) LPS (0.5 μg/ml for 6 h), (iii) OX-A + LPS (30 min pre-incubation with 0.2 μM OX-A and after 0.5 μg/ml LPS 6 h), (iv) SB334867 + OX-A + LPS (15 min 10 μM SB334867 then 30 min 10 μM OX-A and finally 0.5 μg/ml LPS 6 h).

### Transepithelial Electrical Resistance (TEER) Measurement

TEER measurements, used to measure the cell monolayers permeability (19), were determined using millicell® ERS meter (Millipore Corporation) connected to a pair of chopstick electrodes, according to Srinivasan et al. (20). Caco-2 cells were treated for increasing time intervals as detailed above. At each time point, TEER was recorded. The values were expressed as percent of resistance and normalized to the initial value.

### Measurement of Reactive Oxygen Species (ROS)

ROS formation was assayed using dihydrorhodamine 123 (DHR) as described by Palomba et al. (21). Briefly, using the co-culture system, microglial cells were incubated with 10 μM DHR (20 min) and the differentiated Caco-2 cells were plated on the transwell inserts and treated as detailed above. Finally, microglial cells were analyzed with a Leica DMI6000 fluorescence microscope equipped with a Leica DFC320 cooled digital CCD camera (Leica Microsystems). The excitation and emission wavelengths were 488 and 515 nm, respectively. Images were collected with exposure times of 100–400 ms, digitally acquired and processed for fluorescence determination at the single cell level with Metamorph Imaging Software (Leica MetaMorph © AF). Mean fluorescence values were determined by averaging the fluorescence values of at least 50 cells/treatment condition/experiment.

### Immunocytochemistry

Differentiated Caco-2 cells, seeded on glass coverslips in 24-well plates, were cultured until confluence and finally treated with LPS (0.5 μg/mL, for 6 h) or OX-A (0.2 μM for 30 min) +LPS (0.5 μg/mL, for 6 h) or SB334867 (10 μM for 15 min) +OX-A (0.2 μM for 30 min) +LPS (0.5 μg/mL, for 6 h). After treatment, the cells monolayers were fixed for 20 min with paraformaldehyde (4%, wt/vol), rinsed with PBS and blocked in PBS-containing BSA (2%, wt/vol). The rabbit polyclonal anti-occludin (Abcam, Cat No. 222691) was used as a primary antibody. After 18 h at 4°C, cells were washed and exposed to a fluorescein isothiocyanate-conjugated secondary antibody for 2 h in dark. Stained cells were analyzed with a Leica DMI6000 fluorescence microscope equipped with a Leica DFC320 cooled digital CCD camera (Leica Microsystems).

### Animals

Experiments were performed according to the animal protocol approved by the Institutional Animal Care and Use Committee of the University of Novi Sad, Serbia (Document n° 15-92/3, 04/20/2016). Adult male mice C57BL/6j obtained from Charles River Laboratories (Sulzfeld, Germany) were maintained under a 12 h light:12 h dark cycle and controlled environmental conditions (temperature and humidity), with free access to food and water. Animals were treated according four different conditions: (i) PBS as a vehicle, (ii) LPS [intraperitoneal (i.p.) 3.3 mg/kg, 6 h], (iii) OX-A+LPS (OX-A i.p., 40 μg/kg, 1 h before LPS) and (iv) SB334867+OX-A+LPS (SB334867 i.p., 30 mg/kg, 30 min before OX-A injection and 90 min before LPS). SB334867 is a selective antagonist of OX-1R. Animals were then euthanized under deep pentobarbital anesthesia (60 mg/kg, i.p.), and perfused transcardially with 4% (wt/vol) paraformaldehyde/0.1M phosphate buffer, pH7.4 (PB).

### Immunohistochemistry

The brains and the proximal portion of the small intestine were cut with a Leica CM3050S cryostat into 10 μm-thick serial sections in the coronal plane, collected and processed for immunofluorescence or immunoperoxidase with specific primary antibodies. The following primary antibodies were used: anti-occludin 1:200 (Abcam, Cat No. 222691; rabbit polyclonal), anti- Iba-1 1:1,000 (rabbit anti-ionized calcium binding adapter molecule 1; Wako Chemicals, Germany). The following secondary antibodies were used: biotinylated donkey anti-rabbit 1:100 (Vector Laboratories) for occludin and donkey anti-IgGs Alexa-488 (LifeTechnology). Slides were analyzed and images digitally acquired with a Leica DMI6000 microscope equipped with the Leica DFC320 cooled digital CCD camera (Leica Microsystems).

### Statistical Analysis

Data are expressed as mean ± SEM and were analyzed with GraphPad Prism 6 software, version 6.05 (GraphPad, Inc.). Statistical differences among groups were determined by either Student's *t*-test or two-way ANOVA followed by *post hoc* Bonferroni tests for comparison among means. A level of confidence of *P* < 0.05 was used for statistical significance.

## Results

### OX-A Mitigates LPS-Induced Increase of Intestinal Permeability

**Figure 1:**
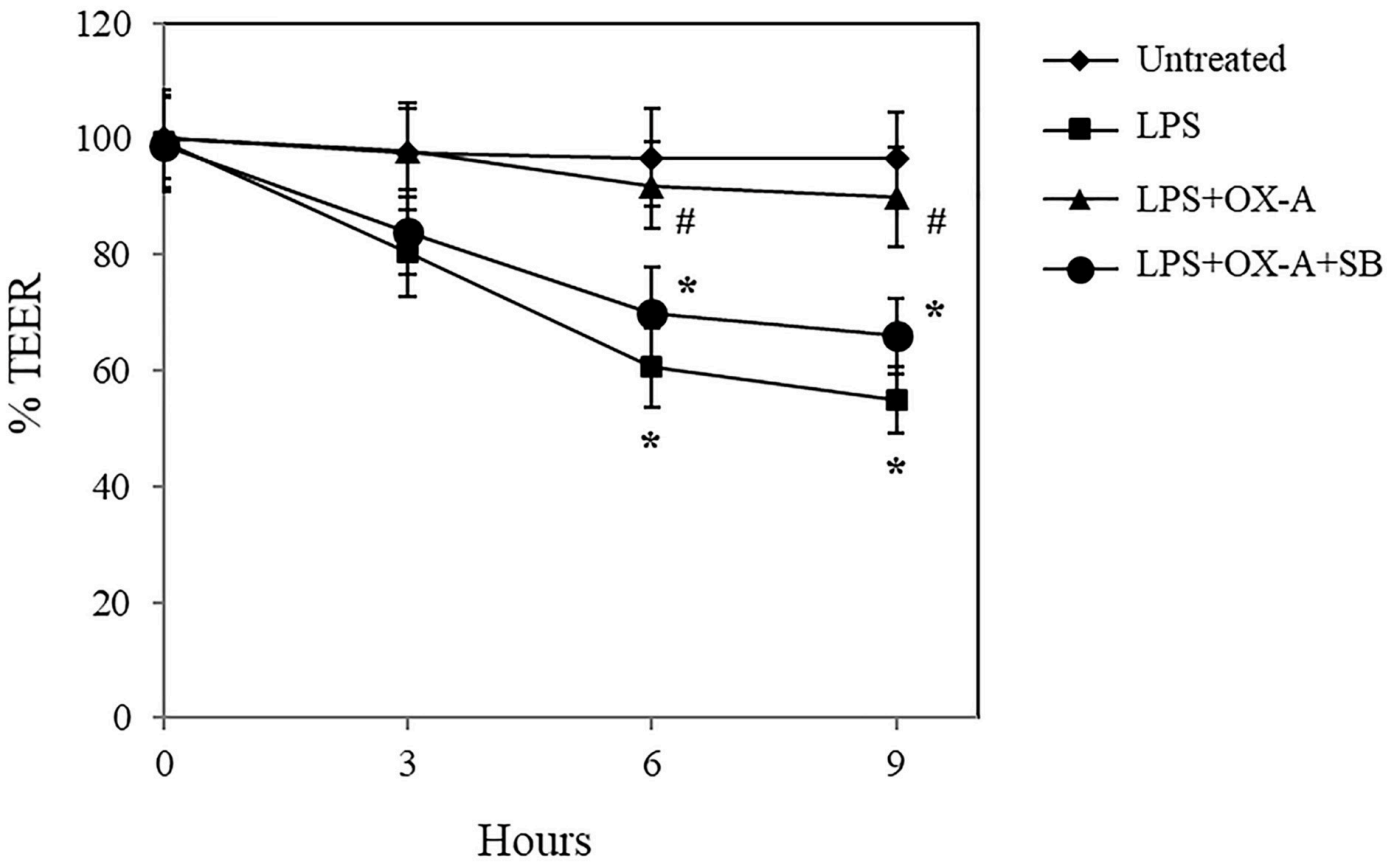
OX-A prevents LPS-induced decrease of transepithelial electrical resistance (TEER) in Caco-2 cell monolayers. Cells were treated for increasing time intervals with LPS (0.5 μg/ml) after a 30 min pre-incubation with OX-A (0.2 μM) with or without SB334867 (10 μM, added 15 min before OX-A). At each time point, TEER was recorded and the values expressed as percent of resistance, normalized to the initial value. Results represent means ± SEM of three separate experiments, each performed in duplicate. *P < 0.01 compared to untreated cells; ^#^*P* < 0.05 compared to LPS-treated cells (one-way ANOVA followed by the Bonferroni's test).

**Figure 2:**
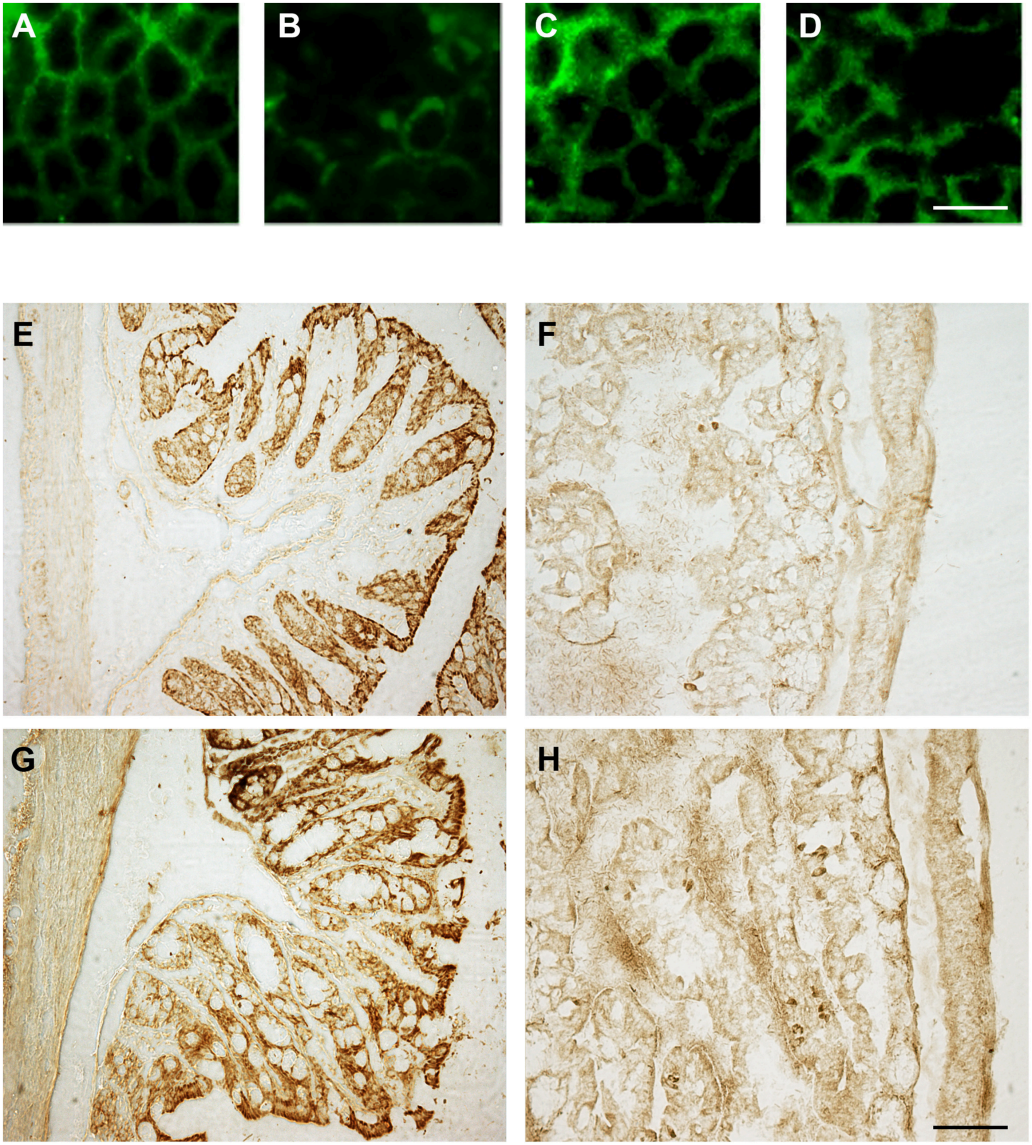
OX-A preserves occludin immunoreactivity in Caco-2 cell monolayers as well as in sections of the small intestine after intraperitoneal administration. **(A–D)** Immunocytochemical staining of occludin (green signal) in Caco-2 cells treated with: LPS (6 h; 0.5 μg/ml) **(B)**; OX-A (30 min; 0.2 μM) +LPS (6 h; 0.5 μg/ml) **(C)**; SB334867 (15 min; 10 μM) +OX-A (30 min; 0.2 μM) +LPS (6 h; 0.5 μg/ml) **(D)**. Untreated cells are also shown **(A)**. **(E–H)** Occludin immunostaining of duodenal epithelial cells in mice treated with: vehicle (saline), **(E)**; LPS (6 h; 3.3 mg/kg) **(F)**; OX-A (1 h; 40 μg/kg) +LPS (6 h; 3.3 mg/kg) **(G)**; SB334867 (30 min; 30 mg/kg) +OX-A (1 h; 40 μg/kg) +LPS (6 h; 3.3 mg/kg) **(H)**. Scale bar **(A–D)**: 20 μm; **(E–H)**: 150 μm.

To investigate the effect of OX-A on LPS-induced increase of intestinal permeability, we used confluent cultures of Caco-2 cells, which express morphological and biological characteristics of small intestinal enterocytes (22), and present OX-1R expression (23). Cells were treated with LPS, OX-A + LPS or SB334867 + OX-A + LPS and intestinal barrier integrity was assessed by measuring TEER, a specific and sensitive marker of intestinal barrier integrity and function (24). LPS treatment (0.5 μg/mL) of Caco-2 cells reduced the basal TEER in a time-dependent manner, reaching 55% of the basal level after 9 h (Figure 1). The treatment with OX-A (0.2 μM added 30 min before LPS) significantly prevented LPS-induced decrease of TEER, resulting in about 90% of the basal level, in a manner prevented with SB334867 (10 μM, 15 min before OX-A addition: 66%), a specific OX-1R antagonist. Occludin, a major component of the TJ protein network, forms a continuous and circumferential structure between the apical and basolateral membrane domains in epithelial and endothelial cells. These proteins, by constituting a controlled diffusion barrier, play a pivotal role in the exchange of substances through the paracellular pathway, and are used as markers of epithelial barrier dysfunction (25). Therefore, to study the effect of OX-A on intestinal barrier integrity, we analyzed the expression of occludin immunoreactivity in Caco-2 cells, after treatment with LPS. Results in Figure 2A show that occludin immunoreactivity in untreated Caco-2 cells appeared as a continuous structure surrounding the cells. This structure was modified by the treatment with LPS (0.5 μg/mL, for 6 h) (Figure 2B), which is known to cause alteration of TJs. Particularly, in LPS-treated cells, the staining of the occludin membrane ring structure was irregular and showed several interruptions. These alterations caused by LPS were prevented by pre-treatment with OX-A (0.2 μM, 30 min before LPS addition) (Figure 2C). The cells in the same condition, treated with SB334867 (10 μM, added 15 min before OX-A), did not show the protective effect of OX-A (Figure 2D).

To confirm also *in vivo* the protective effect of OX-A, we analyzed the immunosignal intensity and distribution of occludin in the proximal portion of the small intestines of treated mice. Immunohistological analysis by optical microscopy of coronal sections of the proximal portion of the small intestine revealed that LPS treatment (3.3 mg/kg, i.p., 6 h) (Figure 2F) strongly reduced occludin immunoreactivity in comparison with vehicle-treated mice (Figure 2E) in a manner prevented by OX-A injection (40 μg/kg i.p., added 1 h before LPS) (Figure 2G) via OX-1R, since the protective effect of the neuropeptide was blocked with an injection of SB334867, a selective antagonist of OX-1R (30 mg/kg, i.p., 30 min before the injection of OX-A) (Figure 2H). These data confirm that OX-A is able to preserve the integrity of the main intestinal tight junction's protein, thus counteracting LPS and pro-inflammatory cytokine translocation into the systemic circulation and endotoxemia.

### OX-A Prevents LPS-Induced Microglia Activation

**Figure 3:**
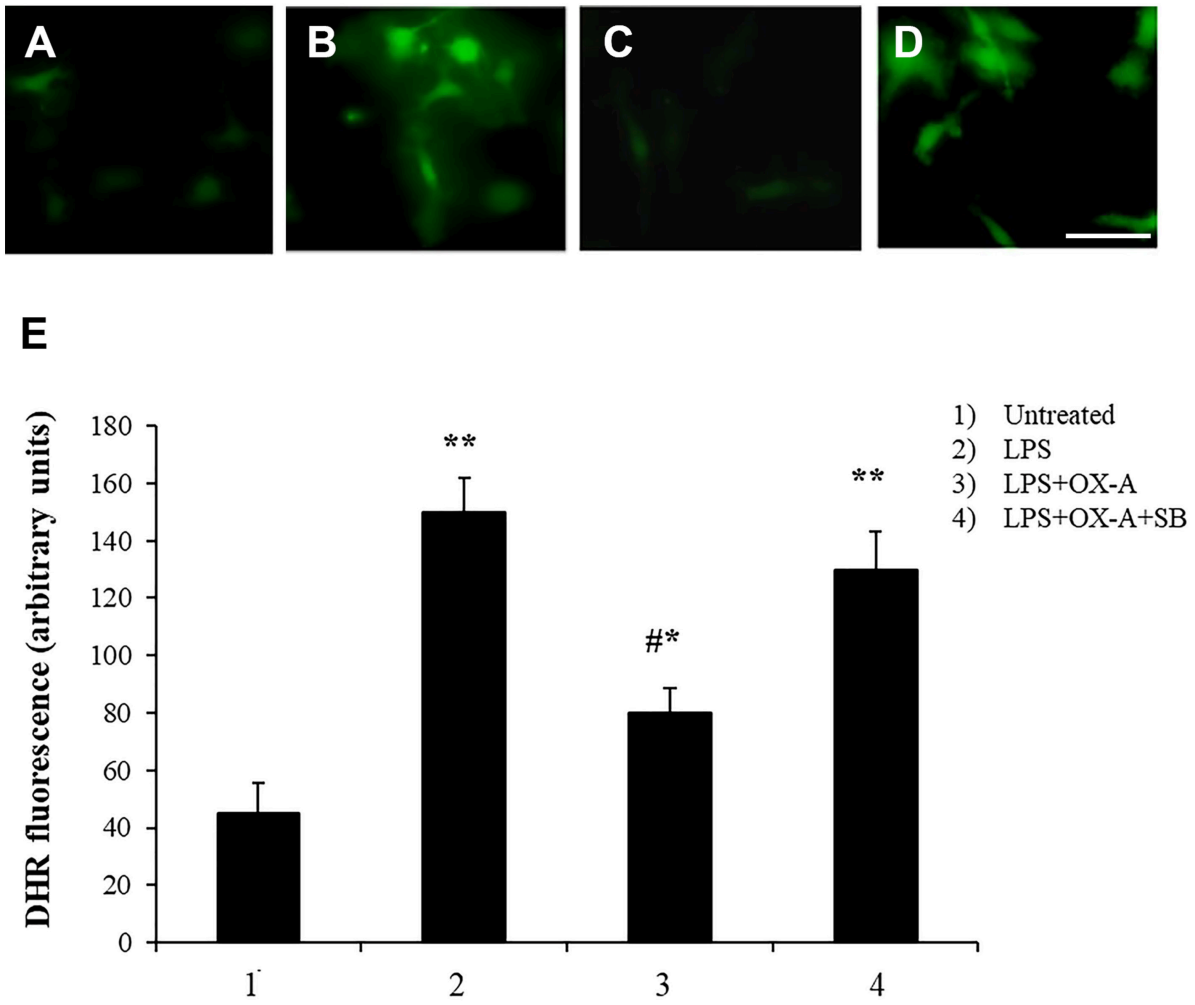
Apical OX-A decreases LPS-induced ROS formation in basolateral primary cultures of microglia. **(A–D)** Representative micrographs of ROS accumulation in primary cultures of microglia co-cultured with Caco-2 as reported in Materials and methods. The transwell insert containing Caco-2 cells was added to the wells containing DHR-loaded microglia and treated in the apical compartment for 6 h with LPS (0.5 μg/ml) **(B)** or with LPS (6 h; 0.5 μg/ml) after a 30 min pre-incubation with OX-A (0.2 μM) in the absence **(C)** or presence of SB334867 (15 min before OX-A; 10 μM) **(D)**. A representative micrograph of control cells is also shown **(A)**. Scale bar: 20 μm. After the treatments, microglia were observed with a Leica DMI6000 fluorescence microscope equipped with a Leica DFC320 cooled digital CCD camera (Leica Microsystems). The resulting images were analyzed to quantify the mean fluorescence of individual cells using Metamorph Imaging Software (Leica MetaMorph AF). **(E)** Graph bars showing DHR fluorescence in primary cultures of microglia co-cultured with Caco-2. Results represent means ± SEM of three separate experiments, each performed in duplicate. *P < 0.05, **P < 0.001 compared to untreated cells; #*P* < 0.001 compared to LPS-treated cells (one-way ANOVA followed by Bonferroni's test).

**Figure 4:**
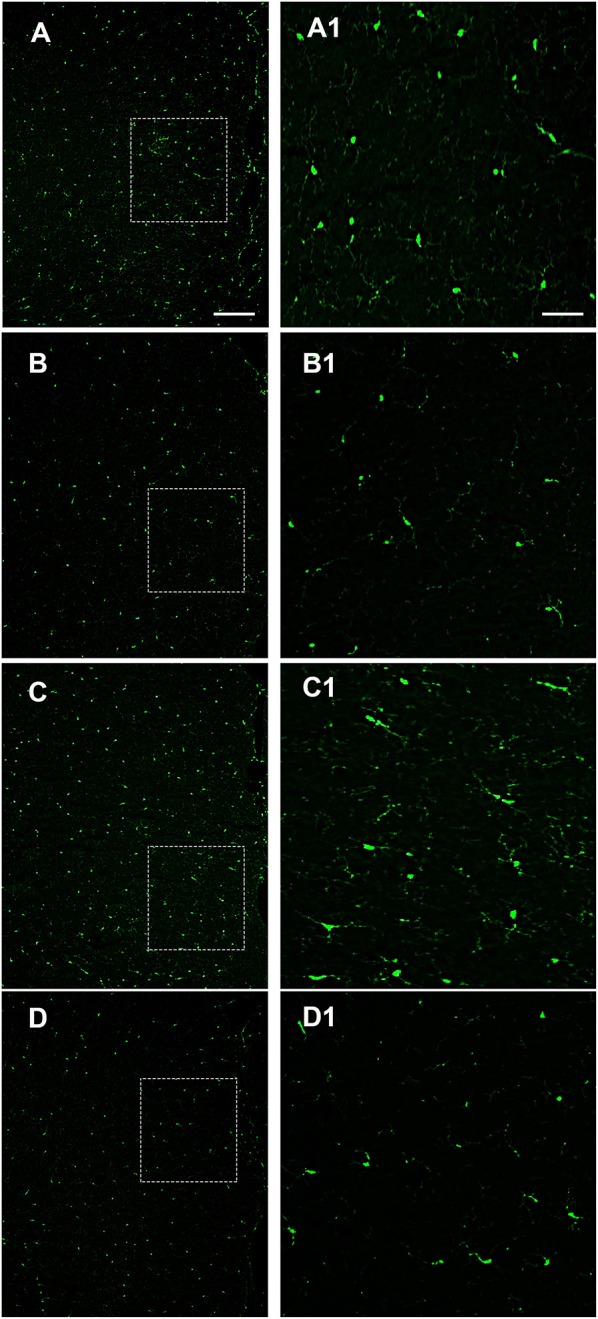
Intraperitoneal administration of OX-A prevents LPS-induced microglia activation in the prefrontal cortex (PFC). Iba-1 immunoreactivity in mice treated with: vehicle (saline) **(A)**; LPS (6 h; 3.3 mg/kg) **(B)**; OX-A (1 h; 40 μg/kg) +LPS (6 h; 3.3 mg/kg) **(C)**; SB334867 (30 min; 30 mg/kg) +OX-A (1 h; 40 μg/kg) +LPS (6 h; 3.3 mg/kg) **(D)**. High magnification of the PFC layers II–III are shown in the boxed area **(A1–D1)** images. Scale bar: 300 μm **(A–D)**, 50 μm **(A1–D1)**.

Alterations of intestinal permeability allow the translocation of inflammatory molecules from the gut to the blood circulation, causing also an impairment of the blood brain barrier (BBB). Considering that microglial cells are the first line of brain defense against endotoxemia, the morphological features of microglia can change by transforming “resting” into “activated” microglia (26). It is well-known that LPS induces ROS formation and consequently microglia activation. We examined a possible protective effect of OX-A against LPS-induced ROS formation and microglia activation. To mimic *in vitro* the connection between gut and brain and to study the putative effect on the cortical microglia, we used a co-culture of Caco-2 cells and primary cortical microglia with Caco-2 cells placed at the apical side of a transwell and primary cortical microglia at the basolateral side. All treatments used to study the apical vs. basal connection were applied to the apical compartment. We used DHR (10 μM, 20 min), a cell-permeable fluorogenic probe useful for the detection of ROS formation, to determine the LPS-induced ROS production and accumulation in the microglia. As shown in Figures 3B and 3E 6 h treatment of apical Caco-2 cells with LPS (0.5 μg/mL) led to ROS production and accumulation in the microglia of the basolateral compartment. This response was reduced by pre-exposure to OX-A (0.2 μM, 30 min before LPS; Figures 3C and 3E), whose protecting effect was abolished by treating the cells also with SB334867 (10 μM, added 15 min before OX-A; Figure 3D). In all these conditions, the size and number of cells were not affected by the treatments compared with untreated cells (Figures 3A and 3E). Next, we sought to investigate if OX-A has a protective effect by preventing microglia activation also *in vivo*. To corroborate this hypothesis, we treated mice with LPS (3.3 mg/kg, i.p., 6 h) and examined Iba-1 immunoreactivity in coronal sections of the prefrontal cortex. LPS injection induced microglial activation in the prefrontal cortex (Figure 4). As shown in Figure 4C, the effect of LPS on microglial activation *in vivo* was reverted by pre-treatment with OX-A under the same conditions that led to protect the occludin network (40 μg/kg, i.p., 1 h before LPS). Indeed, the majority of microglial cells presented a resting phenotype characterized by small cell body and ramified motile processes, typical of their quiescent state found in vehicle-treated mice [Figure 4 compare A (control) and C (OX-A+LPS treated mice) with B (LPS-treated mice)]. This effect was OX-1R-mediated since the injection of SB334867 (30 mg/Kg, i.p., 30 min before the treatment with OX-A, and together with LPS) prevented the beneficial effect of OX-A (Figure 4D). These data suggest that OX-A protects the gut and the brain from alterations of their structural integrity and from the deleterious effect of an enhanced permeability caused by LPS and subsequent metabolic endotoxemia, inflammation and microglia activation.

## Discussion

A dysfunction of the intestinal barrier and the subsequent increase of intestinal permeability facilitate the transit of several substances into the circulation, such as bacteria-derived LPS, thus creating the conditions for systemic and central inflammation. OX-A is a neuropeptide involved in the regulation of energy balance and arousal and can also increase gastric acid secretion after central administration in rats (27). Here we provide evidence of a new potential role of OX-A against LPS-induced decrease of IEB integrity and microglia activation. Therefore, our data suggest that OX-A is one of the players in the gut-brain axis. Indeed, OX-A and OX-1R are present in peripheral tissues, including the top of intestinal villi and intestinal wall (14, 17). Using confluent cultures of Caco-2 cells, a well-known *in vitro* model of small intestinal enterocytes, we demonstrate a protective role of OX-A against LPS-mediated impairment of the IEB. We propose that OX-A is able to prevent LPS-induced decrease in TEER in this *in vitro* model by improving the function of tight junctions. In fact, we showed that treatment with OX-A, by restoring the adequate distribution and amount of occludin, prevents the dysregulation of the intercellular junctional complex induced by LPS. This latter finding was confirmed *in vivo* with the different treatments in mice with LPS in presence or absence of OX-A and SB334867. We hypothesized that these protective effects of OX-A are mediated by the activation of OX-1R localized at the intestinal sections (14, 17), because they were counteracted by the administration of a selective OX-1R antagonist (SB334867) in both *in vitro* and *in vivo* experiments. OX-A, by acting as a protective factor against LPS-induced intestinal barrier impairment, is also likely to prevent the effects of this pro-inflammatory molecule in the CNS. In fact, we also showed that intraperitoneal administration of OX-A was able to reverse the characteristic morphology of activated microglia induced by LPS in the cortex. This protective action was mediated by OX-1R because it was blocked by a selective OX-1R antagonist (SB334867). The protective effect of OX-A was also shown in co-culture experiments with Caco-2 cells and microglia. The apical administration of OX-A was able to prevent the production of ROS induced by LPS in basolateral cortical microglia cells, in a manner also mediated by OX-1R. In agreement with our findings, recent studies have revealed that the peripheral administration of OX-A results in increased survival of mice with LPS-mediated septic shock by inhibiting the excessive production of inflammatory cytokines (28).

**Figure 5:**
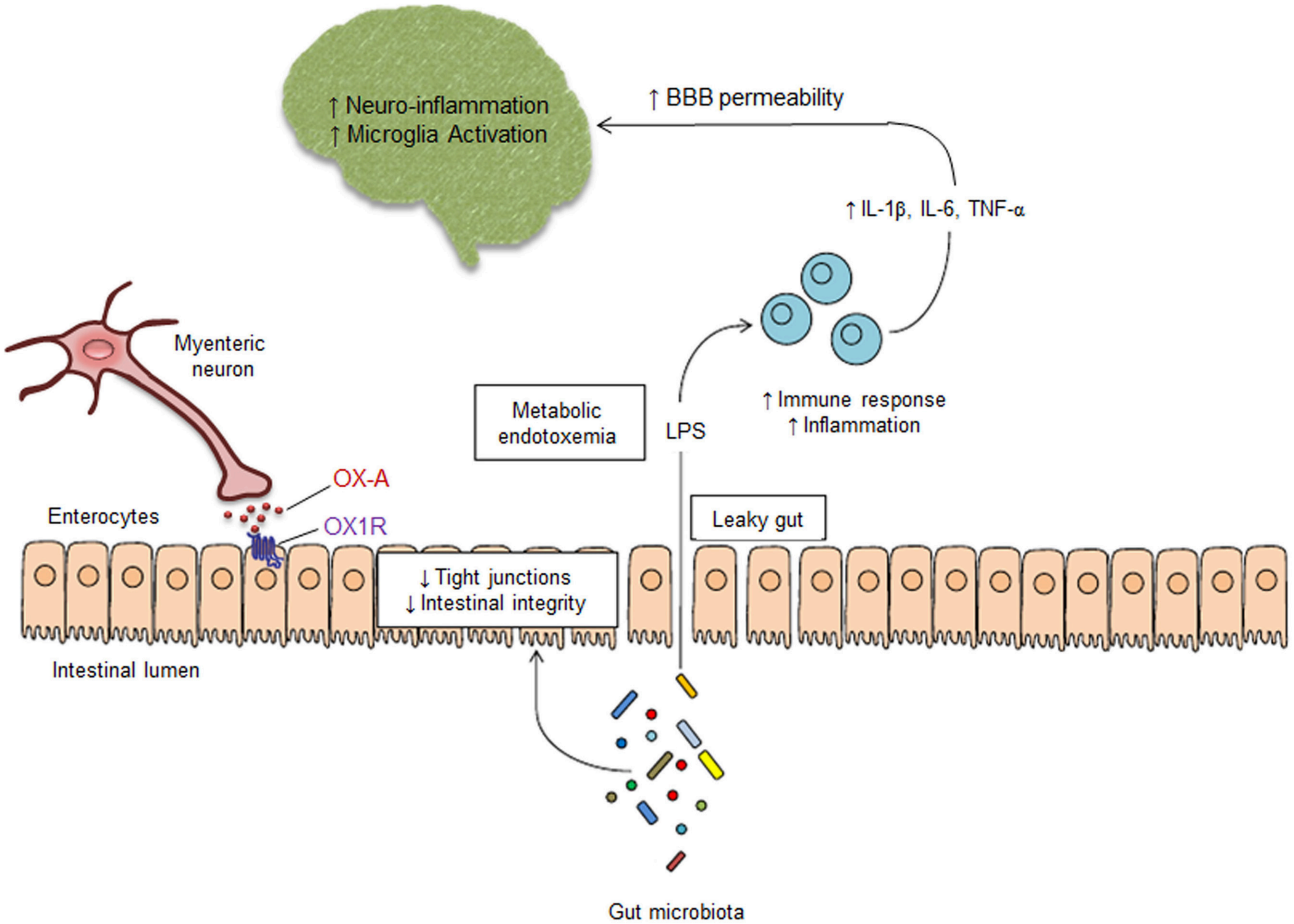
Schematic mechanism of peripheral OX-A-mediated neuroprotection. Alterations of gut microbiota cause the increase of IEB permeability, leading to LPS leaking into the systemic circulation. OX-A released by myenteric neurons, by interacting with OX-1R expressed on the basolateral side of enterocytes, prevents the decrease of occludin expression by the tight junction complex in the intestinal epithelium, thereby preventing intestinal barrier impairment induced by LPS. OX-A blocks the LPS translocation to the blood, thus preventing the metabolic endotoxemia, the activation of immune cells and the subsequent systemic and central inflammation, which are responsible of blood brain barrier impairment and microglia activation. Therefore, peripheral OX-A can act as an epithelial barrier protective factor that may prevent LPS translocation from the gut lumen to the CNS and, consequently, neuroinflammation.

Our present data raise the possibility that OX-A may be released by myenteric neurons to act on OX1-R on intestinal epithelial cells and counteract one of the most important effects of some types of dysbiosis, i.e., the disruption of the IEB induced by LPS (Figure 5). This molecule originated from gram (–) bacteria prevails during conditions such as the prolonged use of antibiotics, unbalanced diets leading to obesity and metabolic disorders, or the chronic consumption of dietary supplements affecting the integrity of the intestinal mucus (29–31). Additionally, since OX-A is increased in the plasma of obese individuals due to excessive release from the lateral hypothalamus (32, 33), our findings also raise the possibility that the effects found in this study may represent a negative feedback mechanism to counteract the increased intestinal permeability and systemic inflammation typical of obesity. Several studies have emphasized the interactions between the CNS and the gastrointestinal system (34–37). The brain levels of a number of neurochemicals important for several neurological functions are under the control of the gut. In particular, some commensal microorganisms play a pivotal role in microglia maturation, differentiation and function (11). We showed here that OX-A, by preventing LPS effects on the IEB, is also able to counteract LPS induction of microglia activation. When activated, microglia secrete, among others, several noxious cytokines and oxygen radicals and sneak between cortical neurons to disrupt synaptic connections, a mechanism known as “synaptic stripping”. We therefore identify a new function for peripheral OX-A as regulator of intestinal permeability and, subsequently, of several effects of dysbiosis, including metabolic endotoxemia and its possible impact on CNS function. Further studies are now needed to investigate whether the mechanisms observed here with exogenous OX-A also occur with the endogenously produced neuropeptide during conditions characterized by dysbiosis and LPS-driven systemic inflammation, such as obesity and ensuing neuroinflammatory pathologies.

## Ethics Statement

Experiments were performed according to an animal protocol approved by the Institutional Animal Care and Use Committee of the University of Novi Sad, Serbia (Document 176; 15-92/3, 04/20/2016).

## Author Contributions

LP and LC conceived and supervised the entire study. LC, VD, and LP designed the experiments. LT, ACF-R, and IM performed most of the *in vitro* experiments and analysis. RC and NM performed the *in vivo* pharmacological treatments. LT, NF, IM, and LD performed the histological preparation and immunohistochemistry. All the authors discussed and analyzed the data. VD and LP wrote the manuscript.

### Conflict of Interest Statement

The authors declare that the research was conducted in the absence of any commercial or financial relationships that could be construed as a potential conflict of interest.

## Abbreviations

BBB: blood brain barrier
BSA: Bovine Serum Albumin
CNS: central nervous system
DHR: dihydrorhodamine 123
IEB: intestinal epithelial barrier
LPS: lipopolysaccharides
OX-A: orexin-A
OX-1R: OX-A receptor-1
PB: phosphate buffer
PBS: phosphate buffer saline
ROS: reactive oxygen species
TEER: transepithelial electrical resistance
TJ: tight junction.
